# The complete mitochondrial genome of *Bombax ceiba*

**DOI:** 10.1080/23802359.2018.1437814

**Published:** 2018-03-05

**Authors:** Yong Gao, Haibo Wang, Chao Liu, Honglong Chu, Yuehui Yan, Lizhou Tang

**Affiliations:** a College of Biological Resource and Food Engineering, Center for Yunnan Plateau Biological Resources Protection and Utilization, Qujing Normal University, Qujing, China;; b Key Laboratory of Yunnan Province Universities of the Diversity and Ecological Adaptive Evolution for Animals and Plants on YunGui Plateau, Qujing Normal University, Qujing, China

**Keywords:** Bombax ceiba, complete mitochondrial genome, Malvaceae

## Abstract

*Bombax ceiba* is a beautiful and deciduous tree with important economic and ecological values. Here, we sequenced the intact mitochondrial genome (mitogenome) of *B. ceiba* on the PacBio sequencing platform (Pacific Biosciences, Menlo Park, CA). The mitogenome is 594,390 bp and is comprised of 35 protein-coding genes, two rRNA genes, and 25 tRNA genes. The phylogeny analysis suggested that *B. ceiba* was closely clustered with the genus *Gossypium*.

*Bombax ceiba* Linn. (Malvaceae), also known as the red silk cotton tree, is a spectacular flowering tree with a height up to 40 m (Barwick 2004). *Bombax ceiba* provides food, fodder, fuel, medicine, and many other economic benefits for natives of many Asian countries (Jain and Verma 2012). And this species is not only useful in economics and medicine but is also an important plant for ecosystem. It is regarded as a pioneer tree species which could survive in low rainfall conditions (Zhou et al. 2015). Despite of the high economic and ecological importance of *B. ceiba*, the genomic information available for this species is still limited. Herein, we sequenced the intact mitochondrial genome (mitogenome) of *B. ceiba* to help with the further molecular studies of this species.

Leaf materials were collected from Yuanmou, Yunnan Province, China (25°40′50.06″ N, 101°53′27.76″ E) in 2017. Genomic DNA extraction was performed with the DNeasy plant mini kit (QIAGEN, Hilden, Germany). Surplus leaf materials as well as the genomic DNA were stored in a −80 °C refrigerator in the Center for Yunnan Plateau Biological Resources Protection and Utilization, Qujing Normal University. The SMRTbell DNA library was prepared and then sequenced with P6, C4 chemistry on a PacBio Sequel instrument (Pacific Biosciences, Menlo Park, CA). With mitogenome sequences of other Malvaceae species as the references, the reads of mitochondrial DNA were filtered from the whole genome sequences of *B. ceiba*. The assembly of the mitogenome was performed with Canu software (Koren et al. 2017). The mitogenome sequence was first annotated on the DOGMA website (http://dogma.ccbb.utexas.edu/) (Wyman et al. 2004), and then manually adjusted. The tRNA genes were annotated with tRNA scan-SE software (Lowe and Eddy 1997).

The mitogenome of *B. ceiba* (GenBank accession MG788014) was assembled into a circular sequence with the length of 594,390 bp, and the GC content was 45.069%. The whole genome was comprised of 62 genes, which included 35 protein-coding genes, two rRNAs, and 25 tRNA genes.

To examine the phylogeny status of *B. ceiba*, mitogenome sequences of nine plants were downloaded from the National Center for Biotechnology Information. Sequences of 20 common protein-coding genes (atp1, atp4, atp6, atp8, atp9, ccmB, ccmC, ccmFC, cob, cox1, cox2, cox3, matR, nad3, nad4, nad4L, nad6, nad7, nad9, and rps3) were aligned by MUSCLE v3.8 software (Edgar 2004). A maximum-likelihood (ML) phylogenetic tree was constructed with Mega v7.0 (Kumar et al. 2016) with the generalized time-reversible (GTR) model and 1000 bootstraps. The phylogenetic tree showed that *B. ceiba* had a close relationship with the genus *Gossypium* (Figure 1).

**Figure 1.**
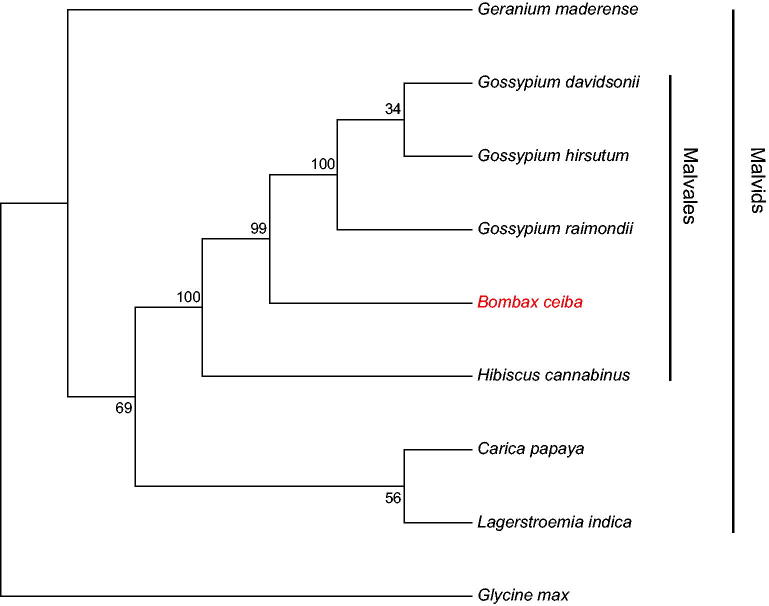
The ML phylogenetic tree constructed with protein-coding genes of mitogenome of *B. ceiba* and other plants. Bootstrap values are shown at each branch. The mitogenome accession number: *Arabidopsis thaliana*: NC_001284.2; *Carica papaya*: NC_012116.1; *Lagerstroemia indica*: NC_035616.1; *Geranium maderense*: NC_027000.1; *Bombax ceiba*: MG788014; *Hibiscus cannabinus*: NC_035549.1; *Gossypium davidsonii*: NC_035075.1; *Gossypium raimondii*: KR736345.1; *Gossypium hirsutum*: NC_027406.1; *Glycine max*: NC_020455.1.
